# Combining Nanomaterials and Developmental Pathways to Design New Treatments for Cardiac Regeneration: The Pulsing Heart of Advanced Therapies

**DOI:** 10.3389/fbioe.2020.00323

**Published:** 2020-04-24

**Authors:** Marco Cassani, Soraia Fernandes, Jan Vrbsky, Ece Ergir, Francesca Cavalieri, Giancarlo Forte

**Affiliations:** ^1^International Clinical Research Center, St Anne’s University Hospital, Brno, Czechia; ^2^Faculty of Technical Chemistry, Institute of Applied Synthetic Chemistry and Institute of Chemical Technologies and Analytics, Vienna University of Technology, Vienna, Austria; ^3^School of Science, RMIT University, Melbourne, VIC, Australia; ^4^Dipartimento di Scienze e Tecnologie Chimiche, Università di Roma “Tor Vergata”, Via Della Ricerca Scientifica, Rome, Italy

**Keywords:** nanoparticles, cardiac regeneration, cardiomyopathy, targeted delivery, Hippo pathway, YAP

## Abstract

The research for heart therapies is challenged by the limited intrinsic regenerative capacity of the adult heart. Moreover, it has been hampered by the poor results obtained by tissue engineering and regenerative medicine attempts at generating functional beating constructs able to integrate with the host tissue. For this reason, organ transplantation remains the elective treatment for end-stage heart failure, while novel strategies aiming to promote cardiac regeneration or repair lag behind. The recent discovery that adult cardiomyocytes can be ectopically induced to enter the cell cycle and proliferate by a combination of microRNAs and cardioprotective drugs, like anti-oxidant, anti-inflammatory, anti-coagulants and anti-platelets agents, fueled the quest for new strategies suited to foster cardiac repair. While proposing a revolutionary approach for heart regeneration, these studies raised serious issues regarding the efficient controlled delivery of the therapeutic cargo, as well as its timely removal or metabolic inactivation from the site of action. Especially, there is need for innovative treatment because of evidence of severe side effects caused by pleiotropic drugs. Biocompatible nanoparticles possess unique physico-chemical properties that have been extensively exploited for overcoming the limitations of standard medical therapies. Researchers have put great efforts into the optimization of the nanoparticles synthesis and functionalization, to control their interactions with the biological milieu and use as a viable alternative to traditional approaches. Nanoparticles can be used for diagnosis and deliver therapies in a personalized and targeted fashion. Regarding the treatment of cardiovascular diseases, nanoparticles-based strategies have provided very promising outcomes, in preclinical studies, during the last years. Efficient encapsulation of a large variety of cargos, specific release at the desired site and improvement of cardiac function are some of the main achievements reached so far by nanoparticle-based treatments in animal models. This work offers an overview on the recent nanomedical applications for cardiac regeneration and highlights how the versatility of nanomaterials can be combined with the newest molecular biology discoveries to advance cardiac regeneration therapies.

## Graphical Abstract

Learning from heart development and diseases to develop novel nano-therapeutics.

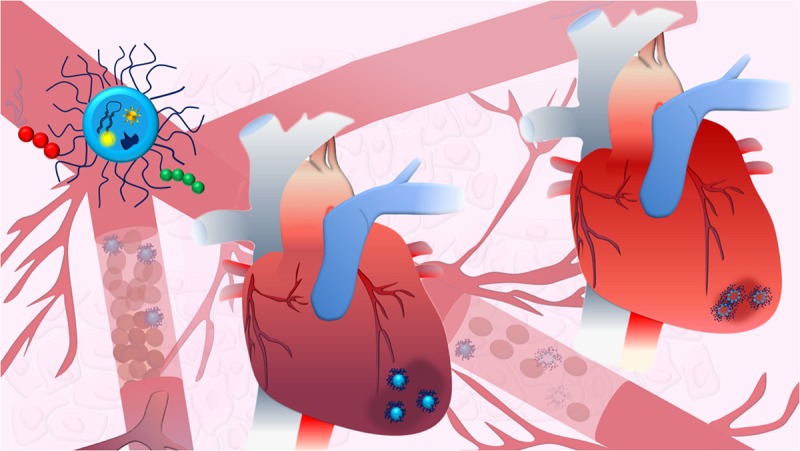

## Introduction

For the last decades, cardiologists and researchers in the field have been fascinated by the idea of treating cardiomyopathies by inducing adult cardiomyocytes (CMs) to proliferate and generate new contractile force (Hashmi and Ahmad, 2019). The regenerative potential of mammalian heart is an age-dependent process and is already limited in newborns (Porrello et al., 2011b). After a few studies reported the limited potential of CMs to regenerate in human hearts during physiological aging and after injury (Bergmann et al., 2009; Senyo et al., 2013), a consensus was recently reached that their capacity is insufficient to restore heart function in case of injury (Eschenhagen et al., 2017). Also, cardiac muscle regenerative potential remains elusive due to the poor understanding of the biology of resident progenitor cells (Tzahor and Poss, 2017).

When damage occurs, rather than producing new functional muscle mass, the human heart is prone to protect its integrity by depositing a non-compliant scar, while inducing cardiomyocyte hypertrophy. Consequently, these two processes lead to the insurgence of arrhythmias and eventually to heart failure (HF). Therefore, overcoming this limitation would revolutionize the good clinical practice by finding a measure to counteract HF (Foglia and Poss, 2016).

To date, several clinical trials have been proposed to test cardiac repair stimulation in adults. However, no satisfactory outcomes were achieved (Banerjee et al., 2018), mainly due to either the poor understanding of resident cardiac progenitor (CPCs) biology in adult heart or by the lack of appropriate delivery tools (Smith et al., 2014; Vicinanza et al., 2017; Cianflone et al., 2018; Marino et al., 2019).

The proposed therapies entailed the transplantation of CPCs or the application of human induced pluripotent stem cells (hiPSCs), mainly delivered through cell injections (Bartunek et al., 2017; Butler et al., 2017), cell-matrix inoculation (Traverse et al., 2019; U.S. National Library of Medicine, 2019a), cell sheets (Miyagawa et al., 2017) and cell patches (Dolan et al., 2019). Despite the amount of work done in this direction, the lack of robust pre-clinical mechanistic studies remains the main hurdle for the failure of cardiac treatment (Menasché, 2018).

In this context, the design of nanoparticles (NPs) targeting the contractile component of the heart may offer interesting solutions to overcome the limitations of current therapeutics, by selective modulation of developmental pathways in cardiac cells. Currently, nanoparticles properties can be tuned and designed opportunely for different medical applications, thus offering the possibility for loading and delivering different kinds of cargos, according to the desired therapy. In this work, the current state-of-the-art of NP-based system for cardiac therapy and their therapeutic cargos such as microRNAs (miRNAs), cardioprotective drugs or growth factors is reported and critically discussed.

In conclusion, we point at Hippo pathway, a recently discovered intracellular axis being involved in fetal heart growth and cardiomyocyte proliferation (von Gise et al., 2012; Heallen et al., 2013), as a promising target for nanoparticle-based therapies.

## The Biology of Cardiomyopathies

Heart failure is either determined by a primary cardiac event, such as in myocardial infarction (MI), or is chronically reached over a long time in non-ischemic cardiomyopathies (Tanai and Frantz, 2015). The treatment options for MI range from anti-inflammatory, anti-coagulants and analgesic drugs to angioplasty, coronary bypass or electronic implants, up to heart transplantation in the most severe cases (Lu et al., 2015). However, if the ischemic event persists for prolonged period, the damage to the heart muscle can be irreversible, and cardiac remodeling, achieved by myocardial fibrosis, results in impaired cardiac function (Briceno et al., 2016). Non-ischemic cardiomyopathies refer, instead, to muscle diseases affecting heart size, shape and structure, that eventually reduce the pumping function of the organ (Chan et al., 2018). They – in fact – proceed to heart blood pump dysfunction, followed by the consequent remodeling of cardiac structures and eventually heart failure (Vikhorev and Vikhoreva, 2018). Based on structural and functional heart changes, several types of cardiomyopathies, having a non-ischemic basis, can be identified. Among them, hypertrophic (HCM), dilated (DCM), restrictive (RC), and arrhythmogenic right ventricular (ARVC) cardiomyopathies are inheritable, and caused by mutations in a single gene (Braunwald, 2017).

**FIGURE 1:**
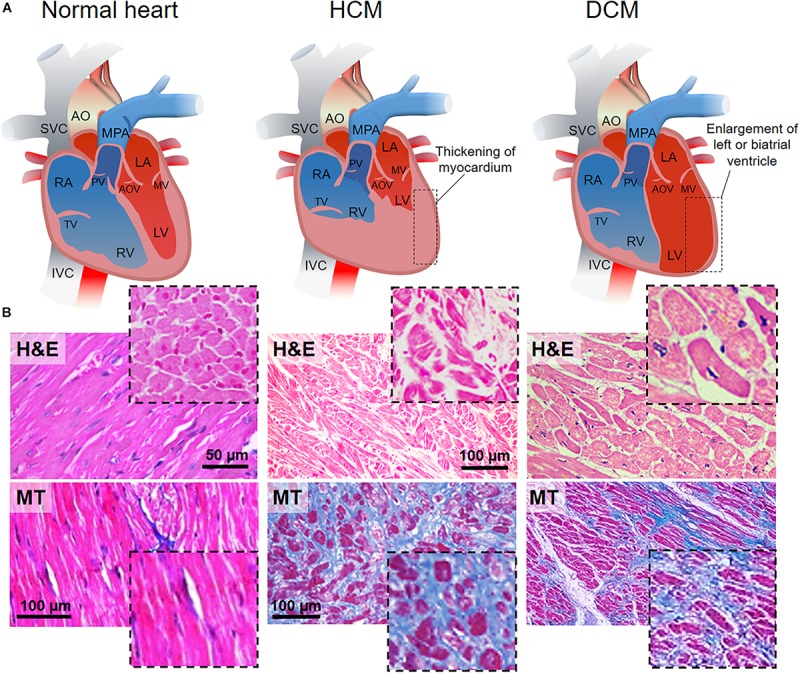
HCM and DCM as the most common cardiomyopathies. **(A)** Schematic representations of the anatomy of normal (left), hypertrophic (hypertrophic cardiomyopathy, HCM, middle) and dilated (dilated cardiomyopathy, DCM, right) heart. HCM is characterized by left ventricle thickening, while DCM is defined by the dilation of the left ventricle. AO, aorta; SVC, superior vena cava; MPA, main pulmonary artery; RA, right atrium; RV, right ventricle; TV, tricuspid valve; PV, pulmonary valve; LA, left atrium; LV, left ventricle; MV, mitral valve; AOV, aortic valve; IVC, inferior vena cava. **(B)** Hematoxylin-eosin (H&E, top) staining in longitudinal and transversal (inset) sections of the cardiac muscle. Normal heart shows the organized and parallel alignment of cardiomyocytes (dashed black line) with preserved cell body and sarcomeric structures. Disorganized cellular structures are highlighted in HCM and DCM sections. Nuclei appear in violet, cytoplasm in pink. Masson’s trichrome (MT, bottom) staining in longitudinal and transversal (inset) sections of cardiac muscle. The homogeneity, the continuity and organization of the healthy tissue (pink) is perturbed by the accumulation of fibrotic non-contractile tissue (blue) in HCM and DCM hearts. Missing scale bars were not provided in the original article. H&E healthy: Reprinted and adapted from Marian and Braunwald (2017) under the terms of the Creative Commons Attribution License. MT healthy: Reprinted and adapted from Wang et al. (2014) under the terms of the Creative Commons Attribution License. H&E HCM: Reprinted and adapted from Jain et al. (2017) under the terms of the Creative Commons Attribution License. MT HCM: Reprinted and adapted from: Guo et al. (2017) under the terms of the Creative Commons Attribution License. H&E DCM: Jain et al. (2017) under the terms of the Creative Commons Attribution License. MT DCM: Reprinted and adapted from: Mitrut et al. (2018) under the terms of the Creative Commons Attribution License.

HCM is caused by single mutations on different specific genes encoding for proteins from the cardiac sarcomere and it is transmitted as a dominant trait (Alfares et al., 2015). Thus, direct relatives of affected people have 50% probability of acquiring the disease. Nevertheless, due to its incomplete penetrance at very young ages, diagnosis of the disease might be delayed till adulthood (Velzen et al., 2018). HCM is characterized by an inappropriate left ventricular hypertrophy (LVH) developed in the absence of pressure overload or infiltration, and, generally, it results in asymmetric septal hypertrophy, but any LVH pattern can be associated with the disease (Mazzarotto et al., 2019). Similarly, gene mutations can result in very distinct LVH patterns in terms of myocardial fibrosis and susceptibility to arrhythmias. Several genes that bear pathogenic mutations causing HCM have been identified. Among them, the combined cardiac myosin binding protein-C (MYBPC3) and b-myosin heavy chain (MYH7) account for up 50% of the clinically recognized HCM cases (Lopes et al., 2015). Histologically, HCM is defined by interstitial fibrosis, myocyte enlargement and microstructure disarray (Figure 1, middle panel).

DCM is the most common cardiomyopathy and a leading cause of heart failure, transplantation and death (Hershberger et al., 2013). It is caused by the pathological dilation of the left ventricle, followed by progressive contractile failure. It is histologically characterized by cardiomyocyte hypertrophy, loss of myofibrils, and interstitial fibrosis (Figure 1, right panel) (Cahill et al., 2013). DCM is a progressive disease that can originate from various factors (acquired or inherited) such as ischemia, infection, autoimmune disease, collagen vascular disease, toxins and drugs, nutritional deficiency, and genetic disease (Watkins et al., 2011; Schultheiss et al., 2019). Although patients with DCM may be initially asymptomatic, progressive heart failure or arrhythmia are often responsible for sudden death cases. DCM has a diverse array of familial or sporadic genetic causes, where mutations can be found in sarcomeric proteins and other structural protein genes (Burke et al., 2016). Mutations in the titin gene *(TTN)* are the most common causes of pathogenicity (∼25–27.6% of familial and 11.6–18% of sporadic cases) (Schafer et al., 2016). Other frequent mutations affect *MYH7* (Colegrave and Peckham, 2014), tropomyosin a1 chain *(TPM1)* (England et al., 2017), and the genes of cardiac troponins (*TNNT2)* (Hershberger et al., 2009).

ARVC is characterized by a progressive replacement of right ventricular myocardium by fibro-fatty tissue leading to ventricular arrhythmias and sudden cardiac death (Xu et al., 2017). Pathogenic mutations in 13 genes have been identified for patients with ARVC with genes encoding the cardiac desmosome accounting for more than 50% of the cases (Xu et al., 2017). The later include plakoglobin (JUP), plakophilin-2 (PKP2), desmoplakin (DSP), desmoglein-2 (DSG2), and desmocollin-2 (DSC2) (Xu et al., 2017).

Analogously to DCM, RC – the least common of the cardiomyopathies – may result from acquired or inherited predispositions. The most significant inherited mutations include *TNNT2*, troponin I (*TNNI3*), α-actin (*ACTC*), and *MYH7* (Muchtar et al., 2017). RC results in increased myocardial stiffness that ultimately leads to impaired ventricular filling (Muchtar et al., 2017). Usually RC is manifested from infiltrative processes, i.e., sarcoidosis, hemochromatosis and amyloidosis, for which tailored interventions and precise diagnosis are required to reveal the disease cause (Muchtar et al., 2017).

Other acquired disorders such as stress-induced and myocarditis cardiomyopathies have been identified. The first one is defined, according to the World Healt Organization (WHO) classification of cardiomyopathies, as an inflammatory disease often resulting from viral infections (Pollack et al., 2015). Clinically it is manifested by acute heart failure, ventricular arrythmias or cardiogenic shock, being associated with significant rates of morbidity and death (Pollack et al., 2015).

Stress or Takotsubo cardiomyopathy is a reversible disorder associated with transient left ventricular dysfunction and affects predominantly post-menopausal women (Nef et al., 2010). It mimics a myocardial infarction but in the absence of coronary artery occlusion and it is manifested by systolic apical ballooning (Dhulipala et al., 2018).

Currently available treatments for cardiomyopathies are mainly focused on the mitigation of the most severe symptoms instead of solving the pathology and in most cases the only resolutive intervention is the heart transplantation (U.S. Department of Health & Human Services).

Conversely, cardiac regeneration therapy looks for the complete remission of the disease to improve the patient’s life quality, aiming to re-establish the lost heart functionality by stimulating the activity of cardiomyocytes.

## Novel Therapeutic Approaches to Treat Cardiomyopathies

Over the years, different kinds of therapies have been used to repair or regenerate the damaged heart. These include: (1) cell-based therapies (Behfar et al., 2014; Madonna et al., 2016); (2) direct reprogramming of resident cardiac fibroblasts into contractile cells (Fu et al., 2015; Engel and Ardehali, 2018); (3) endogenous cardiomyocyte proliferation induction *via* modulation of cardiomyocyte cell cycle regulators, e.g., the Hippo signaling pathway (Mohamed et al., 2018); (4) gene therapy *via* adeno associated viruses (AAVs) (Chamberlain et al., 2017). Since most of these approaches have been exploited when using nanoparticles for cardiac repair, we will briefly introduce them in the following paragraphs.

### Cell-Based Therapies

Cell-based therapies envision the transplantation of cells to restore cardiac function. Implanted cells need to be able to engraft and differentiate into functional cardiomyocytes *in vivo* (Sanganalmath and Bolli, 2013). Several types of cells including skeletal myoblasts (Gavira et al., 2010), bone marrow-derived cells and mesenchymal stem cells (MSCs), cardiac progenitors and pluripotent stem cells [i.e., human embryonic (hESCs) and human induced pluripotent (hiPSCs) stem cells], have been proposed as suitable candidates for cardiac cell therapies (Psaltis et al., 2010; Müller et al., 2018). Despite the promising *in vitro* results and the beneficial short-term outcomes in *in vivo* tests, controversial evidence regarding long term side effects, like arrhythmias or possible tumor growth due to ineffective differentiation, together with inconsistencies in the reported cell engraftment rate and differentiation, have impaired the translation of such approaches into clinics (Rikhtegar et al., 2019). Nevertheless, different clinical trials featuring the delivery of cells, deemed to be beneficial to the heart, are currently undergoing. Most of these studies are based on the direct injection of different preparations of bone marrow-derived cells and are either in the recruitment phase – with estimated completion dates on May 2020 (NCT02032004) and March 2030 (NCT02503280) (Borow et al., 2019; U.S. National Library of Medicine, 2019b, 2020a) – or with completion dates expected on June 2021 (NCT02438306), January 2023 (NCT02408432) and July 2023 (NCT02962661) (Raval et al., 2018; U.S. National Library of Medicine, 2019c, d). The outcomes of these clinical trials are likely to clarify the potentiality of cell-based therapy and mark the future direction for the application of this technique in cardiac regeneration.

### Direct Reprogramming of Resident Cardiac Fibroblasts

Recently, the possibility that cardiomyocytes can be generated by direct cardiac reprogramming of non-contractile cells has gained momentum (Isomi et al., 2019). This approach consists in converting fully differentiated fibroblasts into cardiomyocytes. It combines the beneficial potential of increasing the contractile workforce of the heart with the reduction of the scar tissue formation (Ieda et al., 2010). The protocols currently in use utilize the forced expression of cardiac-specific transcription factors (e.g., GATA4, HAND2, MEF2C, TBX5) (Song et al., 2012) and relevant cardiac miRNAs to hijack the genetic program of non-contractile cells (Jayawardena et al., 2012). Several studies, both *in vitro* and *in vivo*, have reported the use of different combinations of transcription factors, miRNAs or chemical compounds to engineer mouse or human cardiac fibroblasts into cardiomyocyte-like cells, proving functional improvements in MI models (Nam et al., 2013; Wada et al., 2013; Cao et al., 2016). However, problems like inadequate reprogramming efficiency, uncertainty of the molecular mechanisms involved, and the heterogeneous population of induced cardiomyocyte-like cells still need to be addressed before the clinical application of this methodology can be foreseen (Engel and Ardehali, 2018). More details on the use of transcription factors used in cardiac repair have been reported in the literature (Hashimoto et al., 2018).

In the following paragraph, we will describe the role of miRNAs, growth factors and other cardioprotective drugs, in connection with their use as cargos in nanoparticle-driven cardiac regeneration.

### Endogenous Cardiomyocytes Proliferation Induction

#### miRNA Regulation

Because of their regulatory role in cell fate, miRNAs are considered interesting molecular tools and new potent drugs for a number of diseases (Raso and Dirkx, 2017). miRNAs are small endogenous non-coding RNAs (∼23 nucleotides) that play gene-regulatory roles in plants and animals by directing the post-transcriptional repression of protein-coding mRNAs (Bartel, 2009). Over the last two decades, different cardiac miRNAs have been described. Here we refer to heart-specific microRNAs known together as myomiRs (myo = muscle + miR = microRNA), such as mir-128, miR-19a/19b, and other miRNAs with known effects on the heart. They were recently found to play a role in pivotal cell functions involved in cardiac regeneration, i.e., proliferation, reprogramming and differentiation (Huang et al., 2018; Gao et al., 2019).

Additionally, high throughput screening analysis recently performed by different laboratories identified a handful of miRNAs (Table 1) able to induce cardiomyocyte proliferation and stimulate cardiac regeneration in mice and rats (Giacca and Zacchigna, 2015). Following on these studies, miRNAs have also been found to be involved in the cardiac regulation of Hippo pathway, an evolutionarily conserved signaling pathway known for its role in proliferation and apoptosis control during organ development (Meng et al., 2016). This pathway and its impact on cardiac regeneration are described later in this review.

**TABLE 1:** Cardiac function associated miRNAs with references to heart human orthologs.

**MyomiRs gene family**	**Stem-loop sequence (human orthologs)**	**Mature sequence (human orthologs)**	**Heart linked function**	**Cardiac pathology associated function**
mir-1	hsa-mir-1-1	hsa-miR-1-3p	Regulators of cardiac muscle growth and differentiation (Zhao et al., 2005, 2007; Liu et al., 2007, 2008; Callis et al., 2009; Sluijter et al., 2010).	Depletion induces myocyte hyperplasia (Zhao et al., 2007)
	hsa-mir-1-2	hsa-miR-1-3p		
mir-133	hsa-mir-133a	hsa-miR-133a-3p		Reduced in patients with hypertrophic cardiomyopathy (Carè et al., 2007)
mir-208	hsa-mir-208a hsa-mir-208b	hsa-miR-208a-3p hsa-miR-208b-3p		Possible cardio protective effect of miR-208 inhibition in heart failure patients (Kakimoto et al., 2016)
mir-499	hsa-mir-499a	hsa-miR-499a-5p		N.A.
mir-21	hsa-mir-21-5p	N.A.	Enhancement of fibroblast survival, interstitial fibrosis and consequent myocyte hypertrophy (Thum et al., 2008)	N.A.
mir-15	hsa-mir-15a	hsa-miR-15a-5p	Persistence of CM mitosis beyond the normal development window of cell cycle arrest and prolonged cellular proliferation of mouse CMs (Porrello et al., 2011a; Botting et al., 2012)	N.A.
	hsa	hsa		
	hsa	hsa		
	hsa	hsa		
mir-497	hsa-mir-497	hsa-miR-497-5p		
mir-126	hsa-mir-126	hsa-mir-126-5p	Embryonic heart development (Fish et al., 2008)	N.A.
		hsa-mir-126-3p		
mir-128	hsa-mir-128-1	hsa-miR-128-1-5p	Regulator of cell cycle-related genes (Huang et al., 2018)	Deletion promotes cardiac regeneration in adults by activating CM proliferation (Huang et al., 2018)
mir-19	hsa-mir-19a hsa-mir-19b	hsa-miR-19a-5p hsa-miR-19b-1-5p	Cardiac protection-mediated expression induced in heart failure (Gao et al., 2019)	Enhancement of cardiomyocytes proliferation in response to cardiac injury (Gao et al., 2019)
mir-138	hsa-mir-138-1 hsa-mir-138-2	hsa-miR-138-5p hsa-miR-138-5p	Required to establish appropriate chamber-specific gene expression pattern, contributes to CM maturation in zebrafish (Morton et al., 2008)	N.A.
mir-143	hsa-mir-143	hsa-miR-143-3p	Chamber morphogenesis, heartbeat (Miyasaka et al., 2011)	N.A.
mir-195	hsa-mir-195	hsa-miR-195-5p	Ventricular hypertrophy-regulated miRNA (van Rooij et al., 2006)	N.A.
mir-218	hsa-mir-218-1	hsa-miR-218-5p	Heart patterning during embryonic development (Chiavacci et al., 2012)	N.A.
mir-302	hsa-mir-302a	hsa-miR-302a-3p	Heart specific miRNA found in human tissue screening (Lee et al., 2008; Tian et al., 2015)	N.A.
	Has	hsa		
	Has	hsa		
mir-367	hsa-mir-367	hsa-miR-367-5p hsa-miR-367-3p	Heart specific miRNA found in human tissue screening, contributes to proliferation of mouse cardiomyocyte (Tian et al., 2015)	N.A.
mir-486	hsa-mir-486-1	hsa-miR-486-5p	Embryonic heart development via PI3K/Akt signaling (Small et al., 2010)	N.A.
mir-25 (mir-92a family)	hsa-mir-25	hsa-miR-25-3p	N.A.	Inhibition improves cardiac contractility in the failing human heart by boosting intracellular calcium handling (Wahlquist et al., 2014)
				Inhibition of miR-25 in mouse reactivates Hand2 that is crucial for embryonic heart development (Dirkx et al., 2013)
	hsa-mir-92a	hsa-miR-92a-3p	N.A.	Reduces endothelial inflammation and promotes angiogenesis and functional recovery in ischemic myocardium (Bonauer et al., 2009; Loyer et al., 2014)
mir-34	hsa-mir-34a	hsa-miR-34a-5p	N.A.	Anti-apoptotic and telomere protective effect after MI in mice (Boon et al., 2013)
mir-199 mir-590	hsa-mir-199a-1 hsa-mir-590	hsa-miR-199a-3p hsa-miR-590-5p hsa-miR-590-3p	N.A.	Upregulation in rodent heart upon myocardial infarction re-induces mitosis that helps to preserve cardiac function (Eulalio et al., 2012)

**N.A., not assessed.**

#### Growth Factors

Biologically active compounds (i.e., growth factors, cytokines) that act at different levels of regulatory processes can be exploited for cardiac repair. They can, for example, (1) activate resident progenitors to attract and differentiate them at the injury site, (2) induce cardiomyocyte dedifferentiation and proliferation, or (3) induce circulating progenitor cells to trigger neovascularization (Hastings et al., 2015). Unfortunately, growth factors and cytokine have usually pleiotropic effects and very short half-life *in vivo*.

A number of growth factors has been recently conjugated to nanoparticles for a controlled delivery at the heart (Table 2). Vascular endothelial growth factor (VEGF) and fibroblast growth factor (FGF) are among the most potent regulators of neo-vascularization and their efficacy has been tested in pre-clinical applications for improving cardiac function after heart failure (Unger et al., 2000; Simons et al., 2002; Henry et al., 2003). However, neither of them has been yet successfully used in clinical practice (U.S. National Library of Medicine, 2020c; Taimeh et al., 2013), due to their pleiotropic effects and limited half-life *in vivo* (Epstein et al., 2001; Henry et al., 2001).

**TABLE 2:** Drugs and small molecules as cardioprotective agents in nanomedicine applications.

**Molecule**	**class**	**Properties**	**Heart repair properties**	**Clinical trial**	**References**
Simvastatin	Statin	Reduces LDL-C levels	Reduced cardiovascular morbidity and mortality in high risk patients	Heart failure	Heart Protection Study Collaborative and Group, 2002; Feringa et al., 2006; Cannon et al., 2015
VEGF	Growth factor	Promoting neovascularization	Enhanced angiogenesis	Ischemic heart disease and other cardiac conditions	Henry et al., 2001, 2003; U.S. National Library of Medicine, 2020c; Epstein et al., 2001; Taimeh et al., 2013
IGF-I	Growth factor	Regulates contractility, metabolism, hypertrophy, autophagy, senescence, and apoptosis in the heart.	IGF-1 in cardiomyocytes protects the heart from oxidative stress and promotes functional recovery after MI.	FDA approved drugs: Increlex1 and IPLEX1	Troncoso et al., 2014
AMO-1	Anti-miRNA oligonucleotide	Inhibition of miR-1	Reduce apoptosis of cardiomyocytes	N.A.	Xue et al., 2018
CoPP	Anti-oxidant	Suppresses the inflammatory activity of macrophages by induction of heme oxyenase-1 (HO-1) expression	Reduces adverse heart remodeling by controlling the inflammatory activity of macrophages	N.A.	Bulbake et al., 2017
SB431542	Inhibitor	TGFβ inhibitor	Reduces fibrosis, decreases hyperthrophy and improves cardiac function.	N.A.	Ferreira et al., 2018
CHIR99021	Inhibitor	GSK3 inhibitor	Upregulates Wnt signaling resulting in significant increase of mature cardiomyocyte proliferation.	N.A.	U.S. National Library of Medicine, 2020c
Berberine	Alkaloid	Anti-inflammatory, anti-microbial, anti-diharreal, anti-oxidative, vasorelaxant, cholesterol lowering	Reduces rate of MI	Study showed to improve survival of CHF patients when given oral or intraperitoneal	Zeng et al., 2003; Allijn et al., 2017

**LDL-C, low density lipoprotein-C; CHF, congestive heart failure; Increlex1, mecasermin, a human recombinant IGF-1 analog; IPLEX1, mecasermin rinfabate, a binary protein complex of human recombinant IGF-1 and human recombinant IGBP-3; IGBP-3, insulin-like growth factor binding protein-3; HO-1, heme oxygenase-1; TGFβ, transforming growth factor β; GSK3, glycogen synthase kinase-3. N.A., not assessed.**

Furthermore, insulin-like growth factor I (IGF-1) regulates contractility, metabolism, hypertrophy, autophagy, senescence, and apoptosis in the heart and its deficiency in humans and animal models has been associated with an elevated risk of cardiovascular disorders (Troncoso et al., 2014). More specifically, low levels of circulating IGF have been related to the development of heart diseases in patients diagnosed with ischemic heart (Juul et al., 2002). These evidences on the roles of IGF-1 explain the interest on developing new IGF-1-based treatments for heart repair.

Noteworthy, two drugs have been approved by food and drug administration (FDA) for the treatment of IGF1 deficiency: mecasermin (Increlex1) and mecasermin rinfabate (IPLEX1) (Table 2; Troncoso et al., 2014). Nevertheless, the safety of chronic systemic IGF-1 therapy is still open to debate due to the possibility of severe adverse effects such as cancer risk (Troncoso et al., 2014). In order to solve these problems, scientists have selectively overexpressed IGF-1 in the heart, revealing that IGF-1 in cardiomyocytes protects the heart from oxidative stress and promotes functional recovery after MI (Troncoso et al., 2014).

Despite the promising evidence supporting the use of growth factors for cardiac therapy, the development of delivery strategies able to increase the biocompatibility, the circulation time and the release efficiency of these molecules at the injured site must be considered before foreseeing their clinical translation (Rebouças et al., 2016).

#### Selective Regulation of Hippo Pathway to Promote Adult Cardiomyocyte Proliferation

**FIGURE 2:**
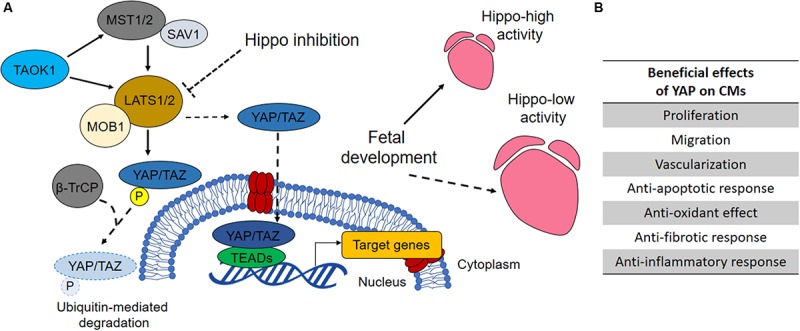
The role of Hippo pathway in heart development and homeostasis. **(A)** When Hippo pathway is on, the mammalian MST1/2 (STE20-like protein kinase 1/2) and SAV1 (protein salvador homolog 1) complex activates LATS1/2 (large tumor suppressor homolog 1/2) that phosphorylates, in association with MOB1 (MOB kinase activator 1), YAP (Yes-associated protein 1) and TAZ (WW domain-containing transcription regulator protein 1), thus promoting their degradation. Conversely, when Hippo signaling is off, YAP and TAZ shuttle into the nucleus where they bind TEADs (TEA domain transcription factor family members) and regulate the transcription of genes involved in cell proliferation, survival and migration (Henry et al., 2001). By regulating the activity of YAP and consequently the proliferation of prenatal cardiomyocytes, Hippo pathway regulates heart size during development. YAP inhibitory kinase TAOK1 (TAO kinase 1) and E3 ubiquitin ligase β-TrCP (β-transducin repeats-containing protein) are involved in YAP degradation. Their silencing has been reported to increase YAP activity in cardiomyocytes (Torrini et al., 2019). **(B)** Increased YAP activity has been associated with several pro-regenerative effects in developed heart to prevent cardiac diseases or promoting restoration after MI, as indicated in the table (Watt et al., 2015).

The Yes-associated protein one (YAP) is the core downstream effector of Hippo pathway. The role of Hippo signaling pathway will be discussed further in the present section. For a more detailed review in cellular mechanobiology, see the review from Martino et al. (2018). The activation of the Hippo pathway results in YAP phosphorylation at Ser127 by upstream LATS kinases (LATS1/2 in human) (Varelas, 2014), which further leads to cytoplasmic sequestration of YAP according to ubiquitin-mediated protein degradation. Conversely, repression of Hippo kinases induces YAP reactivation and accumulation in the nucleus (Boopathy and Hong, 2019) (content Box 1). In the adult organism, YAP is involved in numerous key biological processes where it acts either as repressor or activator in combination with context-specific transcription factors (Figure 2A). From the analysis of almost four hundred direct protein interactors of YAP, it is clear that many biological effects remain unexplored (thebiogrid.org, 2019). Among the most studied functions YAP exerts are stemness maintenance and tumorigenesis (Chen et al., 2019), cell mechanic control via focal adhesions (Nardone et al., 2017) and the regulation of organ size (Watt et al., 2015). The Hippo–YAP pathway regulates heart growth during prenatal life and is considered important for adult heart homeostasis (Figure 2B; Wang et al., 2018). Noteworthy, overexpression of YAP was proven to be sufficient for stimulating proliferation of post-natal rat cardiomyocytes (von Gise et al., 2012). In particular, inducible YAP overexpression in rat embryos and new-born individuals caused an increase in cardiomyocyte proliferation leading to hyperplasia and 20% gain in heart weight in 10 days. This happened due to the increase in cell number, whereas cell size remained unchanged.

**Box 1. Hippo pathway overview.**

Like every tissue of the human body, the heart tissue is subjected to either constant or temporary mechanical stimuli. The cell-extracellular matrix (ECM) interactions dynamically remodel the mechanical properties of the myocardium, and actively respond to extrinsic mechanical cues. **Hippo pathway** is a mechanosensitive signaling pathway transducing external mechanical stimuli into biochemical responses. The pathway functions as a negative regulator of the effectors YAP/TAZ, two paralog proteins acting as transcriptional co-activators. Here are the main components of Hippo pathway and their role in brief: ∙ YAP: Yes-associated protein. The effector of the pathway. It acts as a transcriptional co-activator (Boopathy and Hong, 2019);

• TAZ (WWTR1): WW domain-containing transcription regulator protein 1. Together with YAP, is the effector of the pathway. It acts as a transcriptional co-activator (Boopathy and Hong, 2019);
• LATS1/2: Large Tumor Suppressor Kinase 1. It is a serine/threonine protein kinase directly phosphorylating YAP/TAZ. The phosphorylation inhibits YAP/TAZ translocation to the nucleus (Tang et al., 2019);
• MOB1: MOB Kinase Activator 1A. It functions as a co-factor of LATS1/2 (Kulaberoglu et al., 2017);
• MST1/2 (STK3/4): Mammalian STE20-Like Protein Kinase 2. It acts upstream of LATS1/2 (Qin et al., 2013);
• SAV1 (WW45): Salvador Family WW Domain Containing Protein 1. It forms a heterodimer with MST1/2 (Bae et al., 2017);
• TAOK1: TAO kinase 1. It is a serine/threonine protein kinase acting upstream of MST1/2 (Plouffe et al., 2016);
• B -TrCP: Beta-Transducin Repeat Containing E3 Ubiquitin Protein Ligase (Fuchs et al., 2004);
• TEAD: transcription factor family forming an active transcriptional complex in association with YAP/TAZ (Boopathy and Hong, 2019).

Similarly, blocking the Hippo pathway upstream components MST1/2, LATS2 or SAV1 (WW45) enhanced cardiomyocytes proliferation during heart development (Heallen et al., 2013). Odashima et al. (2007) reported the occurrence of several “pro-regenerative” effects able to inhibit the development of heart failure after myocardial infarction in transgenic mice overexpressing MST1 dominant negative (resulting in downregulation of endogenous MST1 and subsequent YAP upregulation). These effects promoted the reduction of contractile cell apoptosis, intensification of proinflammatory cytokines, inhibition of cardiac dilation, and attenuation of cardiac dysfunction without inhibiting compensatory hypertrophy.

Although progresses in myocardial regeneration in Hippo-deficient heart was reported by Tao et al. (2016), restoration of overall cardiac function by tuning Hippo-pathway components seems to be a more complex task, which requires additional research and validation. In fact, Ikeda et al. (2019) described that long term activation of YAP facilitates the progression of heart failure, in response to pressure overload, in transgenic mice model lacking WW45 Hippo component. Despite homozygous knockout of WW45 (WW45cKO) in mice exhibited greater cardiomyocytes cell cycle re-entry, adverse effects such as interstitial fibrosis, partial increase of infiltrating inflammatory cells and reduction in contractility were also observed.

Considering the very different effects Hippo pathway has on the contractile and structural components of the heart, pros and cons of targeting such a pathway in the whole organ need to be balanced. Also, the crosstalk with other regulatory pathways such as WNT/β-catenin signaling should be considered (Wang et al., 2018). In fact, β-catenin heterozygous mutation (the major effector of WNT pathway) in SAV1 KO mice was able to normalize the proliferation rate of ventricular cardiomyocytes and myocardial thickness, thus confirming the crucial role of WNT pathway in cardiac overgrowth induced by Hippo inactivation (Heallen et al., 2011).

Regarding cardiomyocyte homeostasis, the group of Mauro Giacca lately demonstrated that some miRNAs work in a network that preside over cardiomyocyte homeostasis by converging in the activation of nuclear translocation of YAP (Torrini et al., 2019). In particular, the authors proved that miR-199a-3p, miR-302d, miR-373, miR-590-3p, and miR-1825 can target the TAOK1 and β-TrCP (content Box 1), thus driving E3 ubiquitin ligase-mediated YAP degradation.

These results highlight the crucial role of Hippo pathway in cardiomyocyte homeostasis and the possible cardiac therapy horizons emerging from the regulation of YAP activity in the contractile figures of the heart.

### Adeno-Associated Viruses (AAVs) for Targeted Gene Therapy

Another methodology proposed to treat the failing heart relies on the use of engineered viruses as vectors for transfection, given their natural ability to deliver nucleic acids into replicating host cells (Chen et al., 2017). In this direction, the intra-cardiac administration of miRNA-199a through adeno-associated viral vectors restored contractility and increased pig muscle mass by sustaining cardiomyocyte proliferation and de-differentiation (Gabisonia et al., 2019). Nevertheless, the long-term uncontrolled expression of the miRNA resulted in arrythmia events which led the animals to premature death, most likely due to the proliferation of poorly differentiated cardiac cells.

AAV technology is being used in clinics for several applications. A quick look at the website www.clinicaltrials.gov returns three clinical studies employing AAVs aiming to improving the function of the failing heart in patients with HF with reduced ejection fraction (HFrEF). The studies (CUPID and MYDICAR) relied on the intracoronary or the anterograde epicardial coronary artery infusion delivery of AAV1-encoding sarcoplasmic reticulum Ca^2+^-ATPase (SERCA2a). Although encouraging, with favorable safety profile in terms of immunogenic responses and arrhythmias, the efficacy of the CUPID trial was not confirmed by the larger CUPID 2 study (Penny and Hammond, 2017).

As a general consideration, the use of AAVs still faces important limitations. In particular, several issues remain unsolved, such as (1) long manufacturing processes and scalability; (2) strictly defined cDNA packaging capacity (∼5 kb) that dramatically limits the number of genes that can be carried; (3) the demanding screening of AAV variants suitable for the specific aim; (4) pre-existing immunological sensitivity along with the insurgence of immune response after repeated administrations (Chamberlain et al., 2017).

As an alternative to the use of AAVs, NPs, which can be “custom-made” by using different nano-constructs carrying therapeutic/regenerative drugs/miRNAs, have been proposed, thus opening the way to the application of nanomedicine in the cardiac regeneration field (Amezcua et al., 2016).

## Nanoparticles Design for Cardiac Regeneration

**FIGURE 3:**
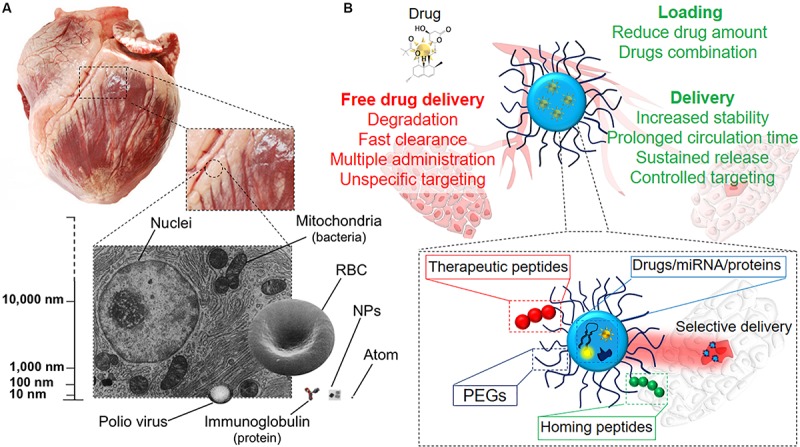
Therapy at the nanoscale. **(A)** Representation of the nanosize, scaling down from the organ size (swine heart) to single cells and intracellular organelles, the latter being the group in which NPs are included. **(B)** Illustrative scheme of the different modalities of drug delivery. NPs as drug delivery systems have plenty of advantages if compared to systemic drug administration, such as targeted release and dosage reduction, thanks to the combination of several functional blocks within the same tool.

Generally, the term *nanomedicine* is applied to a number of innovative therapeutic approaches entailing the use of precisely bioengineered nanostructured materials (Figure 3A), with at least one dimension in the 1–100 nm range (Zhang et al., 2008). However, a broader definition is now accepted for structures above the 100 nm, such as sub-micrometer and nanostructured microparticles, which are commonly regarded as nanomaterials and used for nanomedical applications (Boverhof et al., 2015). Nanomedicine can be defined as the application of nanotechnology to medicine for diagnosis and therapy (Pelaz et al., 2017). It aims to minimize the side effects of therapeutic drugs while increasing their selective accumulation, thus enhancing the efficacy of the treatment in clinics (Davis et al., 2008). Conventional therapies are – in fact – often associated with tremendous side effects due to the intrinsic toxicity of the drugs, their broad spectrum of activity and the poor control over delivery (Jabir et al., 2012). Due to their tunable properties that potentially allow any kind of application, NPs can overwhelm the design limitations associated with AAVs described above. To date, various types of NPs have been loaded with miRNAs and drugs and used to vehiculate therapeutic agents *via* different administration routes, providing several advantages when compared to the standard therapies (Figure 3B). Remarkably, a major limitation in the therapeutic use of miRNAs is their fast clearance and rapid degradation in blood circulation and cellular cytoplasm mainly by ribonucleases, resulting in a short half-life (Sioud, 2005). Furthermore, these molecules cannot freely penetrate into the cell efficiently (Zhang et al., 2007). Extracellular miRNAs are physiologically carried inside the cell by membrane-derived vesicles, lipoprotein and ribonucleoprotein complexes (Boon and Vickers, 2013). Among these systems, exosomes are the main effectors of miRNA carriage and exosome miRNA-loaded release has been found to be involved in intercellular communications (Valadi et al., 2007). Therefore, the use of engineered miRNA nanocarriers represents a nature-inspired approach overcoming the previously described limitations. Several NP-based systems for miRNA delivery were so far developed, as recently reviewed in (Lee et al., 2019).

Along with the miRNA delivery, the use of bioengineered nanocarriers can enhance the circulation time, biodistribution and bioavailability of different drugs and proteins, as well as protecting them from degradation and inactivation (Patra et al., 2018). Indeed, many of the drugs currently available are lipophilic and their systemic administration is challenged by their scarce aqueous solubility, with consequent poor delivery and therapeutic efficiency (Kalepu and Nekkanti, 2015). Consequently, the encapsulation of these molecules inside amphiphilic systems may enhance their efficacy and promote their long lasting and sustained release at the desired site (Din et al., 2017).

Protein therapy offers higher specificity, greater activity, and less toxicity compared to standard drugs. However, the maintenance of their structural complexity and activity, which are crucial for achieving high therapeutic performances, can be challenged by (1) their enzymatic degradation/inactivation, (2) their short circulation half-lives and (3) their poor membrane permeability (Yu et al., 2016). Therefore, the use of nanoparticles may also protect therapeutic proteins from proteolysis while improving their delivery efficiency and sustaining their release at the target site (Zhao et al., 2016).

**FIGURE 4:**
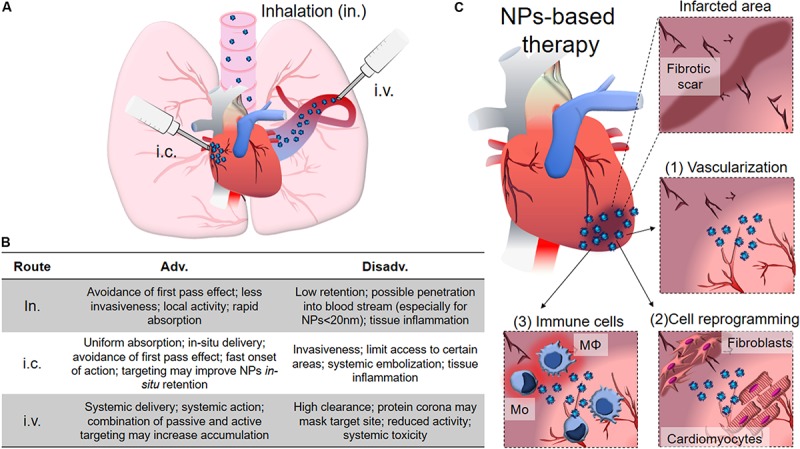
NPs for cardiac diseases. **(A)** The administration routes currently evaluated for delivery therapy at heart include inhalation (in.), intra-cardiac injection (i.c.) and intra-venous injection (i.v.). **(B)** Table showing advantages and disadvantages of the different administration routes. **(C)** The infarcted area typically shows loss of vessels and various degrees of fibrotic scar formation with limited contractility and functionality that dramatically compromise heart functions. NPs can be conceived to help restoring vascularization by promoting angiogenesis (1), locally transdifferentiating fibroblasts into CMs, resolving the fibrotic scar (2), and promoting the anti-inflammatory polarization of immune system cells, like macrophages (Mφ) and monocytes (Mo). The main text provides detailed discussion for each of the points here briefly described.

As a result, the use of nanotechnology to deliver cardioprotective drugs and assist the prolonged release of growth factors has arisen in the last years as a promising tool to restore compromised heart function, as it will be discussed below. Different administration routes, based on the physico-chemical properties of the drug/nanoparticle, on the predicted effect and desired target have been pursued for obtaining an optimal delivery of NPs to treat cardiomyopathies. Intravascular, including intra-cardiac (i.c.) and intravenous (i.v.) injection, and extravascular like inhalation (Figure 4A) are the most common administration routes used for this purpose. They all provide different advantages, and intrinsic disadvantages (Figure 4B; Dib et al., 2011; Yildirimer et al., 2011; Chenthamara et al., 2019), for the treatment of several pathological conditions, such as compromised vascularization, fibrosis and inflammation, while attempting to improve cardiac functionality (Figure 4C). However, despite the encouraging premises, the use of NP-based system for direct cardiac repair is still lagging at the preclinical stage.

In the next chapter we will provide an overview of the state-of-the-art in the field of nanomedicine for cardiac regeneration, and revise the main properties of the NPs – and their cargos – used to this aim (Table 3).

**TABLE 3:** NPs for cardiac regeneration.

**NPs type**	**Composition**		**Cargo**	**Hydrodynamic size and surface charge**	**Targeting moiety**	**Application**	**Stage of research**	**References**
**Polymeric**	DSPE-PEG-NH_2_, DSPE-PEG-Maleimide, PBFT		miR-199a	110 nm, ∼15–20 mV	TAT	Targeted miR-199a delivery for reducing scar size while maximizing muscle and vessel restoration and promote CMs proliferation.	*In vivo*: Rat MI model	Yang et al., 2019
	PEG-DGL		AMO-1	∼200 nm, ∼4 mV	AT1	Targeted delivery of miR-1 inhibitor (AMO-1) to attenuate cardiomyocytes apoptosis.	*In vivo*: C57BL/6 mice MI model	Xue et al., 2018
	HA-sulfate		miR-21	130 nm, -10 mV	N.A.	Delivery of miRNA-21 to cardiac macrophages after MI for inducing their modulation toward an anti-inflammatory, reparative state.	*In vivo*: C57BL/6 mice MI model	Bejerano et al., 2018
	PLGA		Simvastatin	∼160 nm, -4 mV (referred in a different work for similar NPs)	N.A.	Local recruitment of statin-PLGA-NPs-loaded AdSCs to the infarcted site and gradual release of the drug to improve neovascularization and cardiac regeneration.	*In vivo*: BALB/c nu/nu mice	Katsuki et al., 2014; Yokoyama et al., 2019
	PLGA		VEGF	113 nm, -55 mV	N.A.	Local release of reduced dosage of VEGF to favor angiogenesis and reduce risks associated with higher dosage therapy.	*In vivo*: NOD/SCID MI mice	Oduk et al., 2018
	AcDXSp		SB431542 CHIR99021	∼350 nm, ∼10 mV	ANP	pH-triggered delivery of combined poorly water-soluble small drug molecules for promoting cardiac regeneration.	*In vitro*: primary cardiac cells isolated from neonatal rats	Ferreira et al., 2018
	DSPE-PEG-Maleimide, PCPDTBT		N.A.	∼50 nm	CPP	Photoacustic imaging (PAI)	*In vivo*: NOD/SCID mice	Qin et al., 2018
**Liposomes**	HSPC, cholesterol, DSPE-PEG-OH, DSPE-PEG- Maleimide		VEGF	180 nm, N.A.	Anti-P-selectin	Targeted delivery of VEGF to the infarcted site to enhance vascularization.	*In vivo*: rat MI model	Scott et al., 2009
	DSPE-PEG-carboxy, HSPC, cholesterol		N.A.	142 nm, N.A.	AT1	Targeted delivery of NPs (48% accumulation in 24 h) to the left ventricle after MI.	*In vivo*: C57BL/6 mice MI model	Dvir et al., 2011
	PS, PC, cholesterol		N.A.	1.2 μm, -98 mV	PS	Apoptotic cell-like treatment for reducing inflammation at infarcted heart and promoting angiogenesis.	*In vivo*: Balb/c mice MI model	Harel-Adar et al., 2011
**Liposomes**	PMPs		CoPP	100 nm, -2.25 mV	N.A.	Promote biomimicked platelet like proteoliposomes interaction with monocytes, which serve as vehicle for enhanced liposome accumulation at the injured area for local release of therapeutic cargo.	*In vivo*: BALB/c mice	Cheng et al., 2016
	DPPC, DSPE-PEG-OH, cholesterol		Berberine	110 nm, N.A.	N.A.	EPR effect for liposome accumulation and local release of berberine after macrophage uptake, reducing inflammatory damage.	*In vivo*: C57BL/6 mice MI model	Allijn et al., 2017
**Inorganic**	**Core**	**Shell**						
	SiO_2_	IRIS3-APTS	N.A.	50 nm, -25 mV	N.A.	Promoting hMSCs engraftment	*Ex vivo*: Wistar rat infarcted hearts	Popara et al., 2018
	Ca_2_(PO_4_)_2_	Citrate	Hemagglutinin or mimetic peptide	∼200 nm, ∼31 mV	N.A.	Accumulation of nanoparticles at the myocardium via inhalation for local therapy aiming to restore heart contractility.	*In vivo*: Landrace pigs	Miragoli et al., 2018
	Fe_x–__1_O_x__/_SiO_2_	SiO_2_	N.A.	60 nm, N.A.	N.A.	Magnetic nanoparticles internalization on endothelial cells for their guidance to the ischemic heart resulting in improved remodeling and cardiac function.	*In vivo*: rat MI model	Zhang et al., 2019
	Fe_2_O_3_	DMSA, APTs, Glu	N.A.	10/35 nm, -43.1/28.9/-2.1 mV	N.A.	Cardioprotective activity *via* inhibition of intracellular ROS and decrease of peroxidation injury.	*In vivo*: Sprague-Dawley rats and Guinea pigs	Xiong et al., 2015
	Au	PEG-SH, OPSS-PEG-SVA	N.A.	80 nm, N.A.	CNA35	Myocardial scar detection with CT imaging	*In vivo*: Sprague-Dawley rat MI model	Kee and Danila, 2018

**TAT, transactivator of transcription peptide; AT1, angiotensin II type 1 ligand; ANP, atrial natriuretic peptide; CPP, cell-penetrating peptide; AMO-1, Anti-miRNA-oligonucleotide; VEGF, vascular endothelial growth factor; TGFβ inhi, transforming growth factor β inhibitor; GSK3 inhi, glycogen synthase 3 inhibitor; DSPE-PEG, 1,2-distearoylphosphatidyl-ethanolamine-PEG; PBFT, poly(9,9-dioctylfluorene-alt-benzothiadiazole); PEG-DGL, pegylated dendrigraft poly-L-lysine; HA-sulfate, Hyaluronan sulfate; PLGA, Poly lactic-co-glycolic acid; AcDXSp, spermine-acetalated dextran; PCPDTBT, Poly [2, 6- (4,4-bis- (2- ethylhexyl) – 4H -cyclopenta [2,1-b;3,4-b’] dithiophene) –alt -4,7 (2,1,3 – benzothiadiazole)]; AT1, angiotensin II type 1 ligand; CoPP, cobalt protoporphyrin IX; D–PE-PEG, 1,2-distearoylphosphatidyl-ethanolamine-PEG; HSPC, L-α-phosphatidylcholine; PS, phosphatidyl serine; MPs, platelet membrane proteins; IRIS3-APTS, aminopropyltrietoxysilane derivative of IRIS3 cyanine; DMSA, dimercaptosuccinic acid: PEG-SH, PEG-thiol; OPSS-PEG-SVA, orthopyridyldisulfide-polyethylene glycol-succinimidyl valerate; CNA35, collagen binding adhesion protein 35. N.A, not assessed.**

## Polymeric Nanoparticles

**FIGURE 5:**
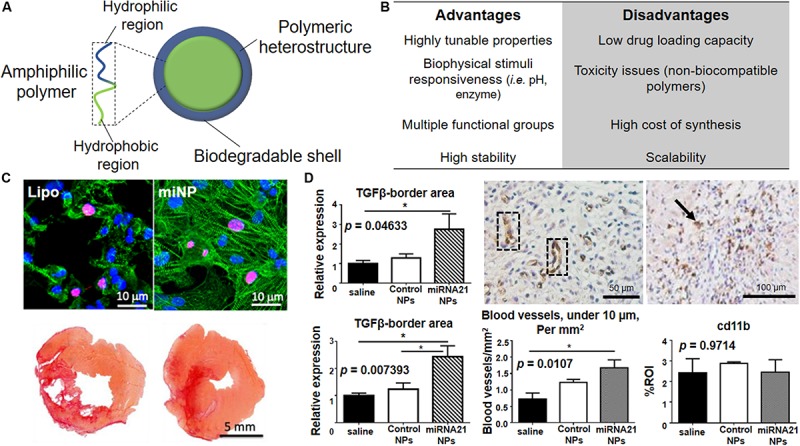
Polymeric NPs for cardiac repair. **(A)** Schematic representation of polymeric NPs with overall composition and structure. The use of amphiphilic building block allows the formation of micelle-like structures in aqueous media. **(B)** Advantages and disadvantages associated with the use of polymeric nanoparticles in term of synthesis, long-term stability and clinical translation of the material. **(C) Top.** Polymeric nanoparticles carrying miRNAs (miNPs) are significantly less toxic for human embryonic stem cell-derived cardiomyocytes when compared to lipofectamine (lipo). Alpha-actinin (green) stains the contractile structure and Ki67 (red) accounts for the proliferation capacity. Nuclei are counterstained by DAPI (blue). **Bottom.** Picro Sirius Red *ex vivo* staining of heart transversal tissue sections showing a clear reduction of the scar size and fibrosis in left ventricle for the heart treated with miNPs (right) in comparison to the same particles carrying a scramble miRNA (left). **(D) Left.** miRNA-21 NPs myocardial infarction treatment causes increased levels of anti-inflammatory cytokine TGF-β both at infarction border zone (top) and in remote myocardium (bottom) when compared to saline-treated or control. **Center.** miRNA-21 NPs induce neovascularization in infarcted mouse myocardium, as shown by CD31 + blood vessels in cross-sections of the heart (dotted black box) and quantified in the graph by comparing it with saline and control NPs-treated groups *p < 0.05. **Right**. Histological analysis shows the presence of macrophages in infarcted heart treated with miRNA-21 NPs, as shown by CD11b-positive cells positive cells (black arrow), but no macrophage accumulation as compared to saline and control NPs as shown in the graph. Reprinted from: **(C)** (Yang et al., 2019) with permission from American Chemical Society; **(D)** (Bejerano et al., 2018) with permission from American Chemical Society.

Polymeric NPs have recently caught attention by virtue of their versatility and higher tunable properties, which make them extremely interesting tools for controlled drug encapsulation and release (Figures 5A,B). Indeed, their physico-chemical properties (i.e., surface charge, surface functionalities, hydrophobicity) can be finely tuned for accommodating nucleic acids, drugs and proteins to promote their efficient release inside the cells (Patil and Panyam, 2009; Fortuni et al., 2019). This large class of NP-based system include amphiphilic micelles, vesicles, dendrimers and polymersomes possessing unique structures and properties, which can be efficiently adjusted during synthesis for hosting different kind of cargos (Chandarana et al., 2018). Most of the designed polymeric NPs propose new synthetic copolymers able to combine different functionalities such as targeting and selective cargo delivery systems (El-Say and El-Sawy, 2017). Moreover, given the emerging use of miRNAs for cardiac regeneration, it is not surprising that many studies developed polymeric NPs as miRNA carriers, alone or in combination with targeting moieties or therapeutic drugs (see Table 2).

Recently, Yang and colleagues have reported the use of polymeric NPs composed by a combination of poly(9,9-dioctylfluorene-*alt*-benzothiadiazole) (PBFT) and 1,2-distearoylphosphatidyl-ethanolamine-PEG-amino (DSPE-PEG-NH_2_), for the conjugation with miRNA molecules, or DSPE-PEG-maleimide for the binding of transactivator of transcription (TAT) peptide, in order to deliver therapeutic miRNA to the infarcted myocardium (Figure 5C) (Yang et al., 2019). The polymeric matrix provided by these miRNA NPs (miNPS) protects miR-199a against enzymatic degradation and facilitates the functionalization with TAT for improved cell uptake. *In vitro* experiments, performed by using hESCs-derived cardiac cells, revealed that the miNPs are less cytotoxic than commercially available lipocomplexes, while exhibiting comparable transfection efficiency, both in normoxia and hypoxia. Interestingly, miNPs were shown to selectively trigger the proliferation of both hESC-derived cardiomyocytes and endothelial cells, but not human cardiac fibroblasts (hCFs). This strategy was able to reduce scar size and maximize muscle and vessel restoration, giving good results *in vivo* when miNPs were injected into mice beating hearts in combination with an injectable hydrogel. Indeed, cardiac function of the neo-vascularized myocardium was restored for over 3 months, indicating the long-term therapeutic effects of this treatment.

In another study the authors pursued targeted delivery of NPs carrying a miRNA inhibitor instead of a therapeutic miRNA. In detail, Xue et al. (2018) developed a pegylated dendrigraft poly-L-lysine (PEG-DGL) dendrimer functionalized with an early myocardium targeting peptide (AT1) and an antisense oligonucleotide able to inhibit miR-1 (AMO-1) (AT1-PEG-DGL-AMO-1). *In vivo* results showed that the nanovector was able to target the infarcted mouse heart within 30–60 min after a single i.v. injection and significantly reduced the infarcted area. The inhibition of miR-1 successfully attenuated cardiomyocytes apoptosis, thus reducing cell death and promoting cardiac repair.

Other than targeting directly cardiomyocytes, immune system cells can be programmed to enhance cardiac repair. The myocardium is – in fact – the site of massive immune cell infiltration during the acute phase of the infarction. In this context, co-assembled miR-21, Ca^2+^ and hyaluronan-sulfate NPs (HASCa^2+^-miRNA) were used to target macrophages in the heart (Figure 5D) (Bejerano et al., 2018). After i.v. injection, NPs promoted the switch of macrophages from the pro-inflammatory to the reparative phenotype, thus promoting angiogenesis while reducing fibrosis and cell apoptosis. However, the poor understanding of the mechanism by which the interaction between NPs and macrophages occurs remains the major obstacle to the translation of this appealing procedure.

By using a different approach, Yokoyama et al. (2019) combined nanomaterials and stem cell therapy. The capacity of adipose-derived stem cell (ADSCs) for promoting neovascularization and inhibiting cell death after MI was exploited. The authors reported that simvastatin-conjugated PLGA NPs loaded *in vitro* on ADSCs induce spontaneous recovery of infarcted myocardium and increase vascularity. Indeed, statin-PLGA-NPs-loaded ADSCs were shown to be recruited to the ischemic myocardium to locally release the payload. This combined approach is bound to increase the cardiac regeneration potential of a very limited number of cells (10,000 cells per mouse), since the effect would be amplified by statin gradual release. This research work interestingly pointed out the therapeutic benefits emerging from the combination of NP-based and cell-based therapies for treating cardiac diseases.

Additionally, Oduk et al. (2018) described the use of PLGA NPs VEGF-loaded to restore the vascularization at the infarcted heart. The particles were able to continuously release VEGF for at least 31 days after injection in a mice model of MI, with improvements in cardiovascular system being still detected 4 weeks after the treatment. Noteworthy, the controlled release of the growth factor, due to the continuous biodegradation of PLGA matrix, effectively increased the delivery of VEGF at the target site, while reducing its systemic side effects.

In the context of cell reprogramming, spermine-modified acetylated dextran (AcDXSp) nanoparticles have been designed to encapsulate poorly water-soluble drugs (SB431542 – transforming growth factor β (TGFβ) inhibitor – and CHIR99021 – Glycogen synthase kinase-3 (GSK3) inhibitor) used to reprogram fibroblasts (Ferreira et al., 2018). These NPs were also tagged with a targeting peptide (atrial natriuretic peptide, ANP) specific for cardiac fibroblasts. The authors claimed the dual targeting and therapeutic effect might be exploited to circumvent the limitations of local injection. Nevertheless, *in vivo* experiments to prove this theory were not carried out so far.

Along with the efforts spent for developing therapeutic nanomaterials, NPs can also be designed as diagnostic tools allowing for superior performance in imaging cardiomyocytes in the failing heart or to monitor the progress of therapeutic protocols. Among the diagnostic techniques available at present, photoacoustic imaging (PAI) is a non-invasive diagnostic tool which provides high sensitivity and helps overcoming the limited depth penetration and spatial resolution of the conventional optical imaging (Wang and Hu, 2012). In order to increase the image contrast when using this technique, nanoparticles have been successfully employed as contrast agent material (Calcagno et al., 2019).

Qin et al. (2018) recently reported the use of NPs composed by a semiconductor polymeric contrast agent (PCPDTBT) encapsulated in a FDA approved lipid-based copolymer (DSPE-PEG-Maleimide). NPs functionalized with cell-penetrating peptide (CPP) were able to target and label hESCs-derived cardiomyocytes (hECS-CM). This labeling technique was suitable for cell detection using PAI both *ex vivo* and *in vivo*. In the latter case, the resolution obtained was as low as 2,000 injected cells, twenty-five times lower than what can be achieved with fluorescent imaging. Photoacoustic imaging was also successfully applied to monitor hECS-CMs transplantation in living mouse heart. Although being able to detect NPs-labeled cells, PAI sensitivity was lower for imaging the host myocardium, as recognized by the same authors. Nevertheless, this strategy holds great promises for monitoring cardiac regenerative processes and for live imaging of heart in the future. However, its application in monitoring cardiac disease and cardiac regeneration is still poorly investigated.

Despite the invaluable properties of polymeric NPs, their toxicity and biodegradability have to be carefully considered during their design. The use of non-biodegradable polymers has been associated with chronic toxicity and, as general warning, the long-term toxicity of these materials is still largely unknown (Banik et al., 2016). Consequently, the use of well-know and established biocompatible polymers is instead more likely to facilitate and improve the clinical translation of polymeric NPs (Ferrari et al., 2018).

## Liposomes

**FIGURE 6:**
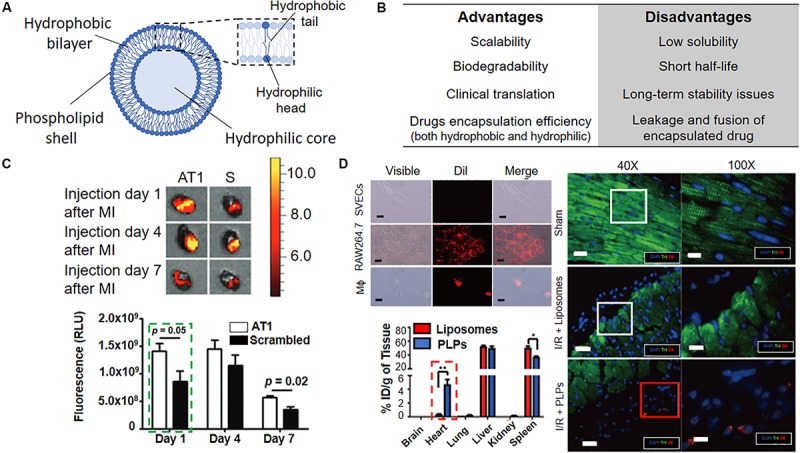
Liposomes for active targeting of infarcted heart. **(A)** Schematic representation of liposomes with overall composition and structure. **(B)** Advantages and disadvantages associated with the use of liposomes. **(C) Top.** *In vivo* bioluminescence images of infarcted mouse hearts injected either with AT1 or scrambled (S) liposomes and analyzed at the indicated timepoints indicate AT1-liposomes preferential accumulation in the tissue (orange/red signal). The graph shows the quantification of the fluorescence due to AT1- and scramble-liposomes accumulation at the given timepoints. **(D) Top left.** Dil-labeled-Platelet-like proteoliposomes (PLPs) selectively interact with RAW264.7 monocytic cell line and macrophages (Mϕ) but not with SVECs endothelial cells, as indicated by the red signal. Scale bar 10 μm. Dil = 1,1′-Dioctadecyl-3,3,3′,3′-Tetramethylindocarbocyanine Perchlorate. **Bottom left.** Intravenously injected PLPs accumulate in the heart more efficiently than liposomes 72 h post-infarction *p < 0.05, **p < 0.01. **Right.** The persistence of Dil-labeled PLPs in the infarction area in a mouse model of ischemia/reperfusion (I/R + PLPs, red square) as compared to Dil-labeled liposomes (I/R + Liposomes, white square) and sham control. Troponin I (TnI, green), stains the heart muscle, Dil (red) injected liposomes, cell nuclei are counterstained with DAPI (blue), Scale bar were not reported in the original article. Reprinted and adapted from: **(C)** (Dvir et al., 2011) with permission from American Chemical Society; **(D)** (Cheng et al., 2016) with permission from Wiley.

Another type of widely studied carrier for drug delivery/transfection applications is represented by liposomes (Figures 6A,B). Liposomes are artificial vesicles of phospholipids and cholesterol mixture, able to encapsulate drugs, proteins/peptides, and DNA (Akbarzadeh et al., 2013). Although they may face long-term stability issues in blood circulation, liposomes are extremely interesting for their cost-effective and scalable synthesis. These features have favored their clinical development (Doxil was the first FDA-approved nanodrug) as compared to other kinds of NPs (Sercombe et al., 2015). One of the first examples of liposomes applied to cardiomyopathy treatment was represented by anti-P-selectin-conjugated liposomes containing VEGF, as reported by Scott et al. (2009). The authors attempted to enhance neovascularization in a rat model of myocardial infarction. Targeted delivery through P-selectin antibody, overexpressed at the infarcted inflammatory site, promoted the selective and efficient delivery of VEGF, thus enhancing the fractional shortening and systolic function, with a 21% increase in anatomical vessels and 74% increase in perfused vessels in MI area. Conversely, systemic administration of VEGF resulted in no significant improvement in cardiac function.

In another study, PEGylated liposomes containing a targeting ligand against angiotensin II type 1 (AT1), a receptor which is widely expressed in infarcted heart were reported (Figure 6C; Dvir et al., 2011). *In vivo* experiments have confirmed particles accumulation in the left ventricle after MI (48% within 24 h post-i.v. injection), thus revealing a specific delivery at injured myocardium. No detail on the functional therapeutic cardiac regeneration potential of the nanovector was given, as the main rationale of the authors was to demonstrate the targeting efficiency of NPs at the heart after MI. Nevertheless, this study provides crucial information for understanding the accumulation of NP-based system at the diseased heart, as discussed below.

The use of liposomes for targeting the immune system cells, which regulates the inflammatory response at the infarcted heart, has also been studied. Targeting the immune system cells at the site of inflammation can be indeed an alternative and effective solution for increasing the targeting and delivery of NP-based therapy, due to the natural tendency of the innate components to recognize and internalize external materials administered in the body such as nanoparticles (Fadeel, 2019). The group of Cohen S. reported the use of phosphatidylserine (PS)-presenting liposomes, mimicking the anti-inflammatory effects of apoptotic cells (Harel-Adar et al., 2011). The uptake of PS-liposome induced the secretion by macrophages of high levels of anti-inflammatory cytokines (i.e., TGFβ and interleukin 10, IL-10) both *in vitro* and *in vivo*. The i.v. injection in a rat model of acute MI promoted angiogenesis, while preserving small scars and preventing ventricular dilatation and remodeling.

More recently, an alternative approach was presented by Cheng and collaborators (Figure 6D; Cheng et al., 2016). The authors developed biomimicking platelet-like proteoliposomes able to interact with monocytes. This methodology foresees the interaction between the immune system cells and platelet membrane proteins (PMPs) for promoting the accumulation of liposomes at the injured heart, where monocytes are recruited. Proteoliposomes were loaded with therapeutic cobalt protoporphyrin IX (CoPP), a compound able to suppress the inflammatory activity of macrophages and were “dragged” by the monocytes to the infarcted zone. This system showed promising results *in vivo* when compared to systemic administration of free CoPP. However, the randomization of the surface coating may jeopardize the reproducibility of this synthetic technique, as raw material, i.e., the PMPs derived from different samples or batches, could give different results in term of composition and biophysical features of the final particles. Moreover, the poor understanding of the interaction with monocytes, which can reduce the effective control over this mechanism, makes the clinical translation of proteolioposomes unlikely.

Furthermore, the group of Schiffelers have developed a liposomal carrier for berberine delivery, a natural product which is known for its anti-inflammatory, anti-oxidative and cardio-protective functions (Allijn et al., 2017). In its free form, berberine is poorly soluble in aqueous medium and have a short half-life time in circulation. The liposomes encapsulated with the dug tested *in vivo* in C57BL/6J mice showed to preserve the cardiac function by 64% at day 28 post-MI, in comparison to control liposomes and free drug. Liposomes administered I.V. targeted the inflammatory site and release the drug after macrophage uptake, reducing both inflammatory damage and systemic adverse effects. This study appears particularly promising because of the reproducible synthesis of the liposome formulation and for its effective targeting and long-term stability.

So far, liposomes have been the most tested NP-based system in clinical trials, encountering several cases of successful clinical translation, and many liposome formulations are currently available on the market for several therapies (Bulbake et al., 2017). However, their application in heart diseases therapy is still limited and needs further investigation.

## Inorganic Nanoparticles

**FIGURE 7:**
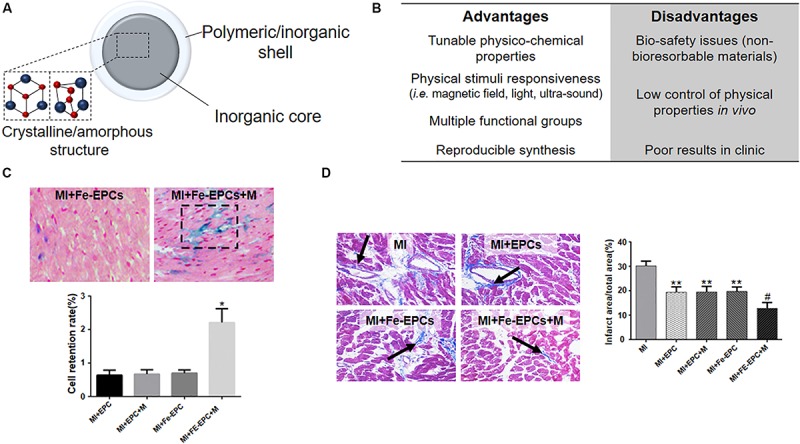
Inorganic NPs for cardiac regeneration. **(A)** Schematic representation of inorganic NPs used for cardiac regeneration. **(B)** Table listing the advantages and disadvantages associated with the use of inorganic nanoparticles. **(C)** Representative hematoxylin/eosin (cell nuclei and cytoplasm) and Prussian blue (iron oxide nanoparticles) images of infarcted myocardium (MI) injected with endothelial progenitor cells loaded with iron oxide nanoparticles (Fe-EPCs) and exposed (+M) or not to external magnetic field. The graph quantifies the retention of EPCs and Fe-EPCs in the presence or not of magnetic field *p < 0.05. **(D)** Representative Masson’s trichrome staining of infarcted myocardium (MI) injected with endothelial progenitor cells loaded (Fe-EPCs) or not (EPCs) with iron oxide nanoparticles and exposed (+M) or not to external magnetic field. Blue color and black arrow identify the fibrotic area. The graph quantifies the infarction area as obtained by image analysis. *p < 0.05 versus the control Sham operated group (not shown), ^#^*p* < 0.05 versus the other group shown in the graph. Reprinted and adapted from: **(C,D)** (Zhang et al., 2019) with permission of Wiley.

Inorganic NPs are known for exhibiting appealing physical properties that can be potentially exploited for simultaneous diagnosis and therapy (i.e. theranostic) of several pathologies (Figures 7A,B; Giner-Casares et al., 2016). They are generally composed by an inorganic core surrounded by an organic/inorganic shell, which aim to increase the biocompatibility of the system and the interactions of the NP with the biological environment (Conde et al., 2014). Recently, Popara et al. (2018) showed that SiO_2_-NPs passively interacted with human MSCs (hMSCs) mediating important molecular processes (Figure 7C; Popara et al., 2018). More specifically, the internalization of the SiO_2_-NPs affected focal adhesions by promoting cell adhesive phenotype both *in vitro* and *ex vivo* upon injection in the infarcted rat heart. In addition, NPs internalization contributed to cell cross-talk between transplanted cells and the host, which is essential for an effective engraftment and tissue regeneration. However, the transient inhibition of lysosomal function by SiO2-NPs was reported, pointing out the need for a thorough evaluation of NPs long-term toxicity and possible side effects due to their sustained intracellular accumulation (Croissant et al., 2017).

Biodegradable inorganic particles have been also proposed to improve myocardial function after heart failure (Miragoli et al., 2018). Miragoli and co-workers demonstrated that biodegradable negatively charged calcium phosphate NPs (CaP-NPs) accumulated at the myocardium 60 min after inhalation in a mice model. The NPs functionalized with a non-penetrating mimetic peptide (NPs-MP) were shown to cross the alveolar-capillary barrier in the lung and translocate to the myocardium where the loaded peptide can be release for therapy. Despite the great potential, there are still some limitations to the use of such therapy. One above all, the mechanisms through which the NPs cross alveolar-capillary barrier are still unclear and unlikely to be accepted for clinical translation.

Interestingly, also the exploitation of hybrid materials, i.e., NPs composed by different inorganic core-shell structures, have been reported. Zhang et al. (2019) described the use of silica-coated magnetic nanoparticles for labeling endothelial progenitor cells (EPCs) for their magnetic guidance at the ischemic heart (Figure 7D), since EPCs are the most used cells for cell therapy after MI due to their mobilization, homing, and angiogenic effects (Zhang et al., 2019). In this study the authors showed the increased retention of EPCs at the infarcted border zone and the consequent attenuation of myocardial apoptosis associated to improved remodeling and cardiac function. Nevertheless, the evidenced improvements only lasted for a very limited term, claiming for the necessity of multiple administrations of the NP-cell system and possibly limiting the advantages obtainable with a NP-based therapy in comparison with other treatments.

Iron oxide nanoparticles have also been investigated as cardioprotective agents, as shown by Xiong et al. (2015). The authors demonstrated the potential of maghemite NPs for protecting the heart from ischemic damage both *in vivo* and *in vitro*. More specifically, Fe_2_O_3_ NPs coated with dimercaptosuccinic acid (DMSA) were demonstrated to efficiently inhibit calcium influx, which is responsible for the reactive oxygen species (ROS) production, therefore decreasing the peroxidation injury of membrane lipids. In addition, these NPs were able to increase the level of S-nitrosothiols and then to participate in nitric oxide (NO)-mediated protection against ischemia and reperfusion injury. However, the mechanisms which regulate the crosstalk between ROS, NO and calcium influx pathways and NPs remain unknown and possible long-term side effects of this activity has not been elucidated.

Diagnostic tools were also developed using inorganic nanoparticles. In particular, gold nanoparticles have been demonstrated to be promising tools for computed tomography (CT), an X-ray-based image diagnostic technique able to exploit the differences in the absorption from different human tissues in order to produce images of body structures and tissues (Xi et al., 2012). In this context, Kee and Danila (2018) reported the use of gold nanoparticles coated with collagen-binding adhesion protein 35 (CNA35) for CT imaging of infarcted heart at molecular level. Thanks to their ability to target collagen I, abundant at myocardial scar, CNA35-Au NPs were able to enhance the signal from the infarcted site at 6 h after injection. Conversely, no detectable enhancement was noted when non-functionalized AuNPs was injected into rats with or without MI or CNA35-AuNPs in control rats without MI. These results highlighted the preferable use of gold nanoparticles as contrast agents compare to iodinated agents in terms of functionalization and blood circulation time, which ultimately could improve the *in vivo* targeting and detection of infarcted heart. However, some issues such as the relatively high amount of nanoparticles required and the inefficiency to enhance the contrast of the entire infarcted area may wane the application of Au NPs for CT-imaging, as stated by the same authors.

Currently, several clinical trials using inorganic nanoparticles are under investigation for applications in cardiovascular diseases (U.S. National Library of Medicine, 2020b). However, with regard to MI, only magnetic resonance imaging (MRI) applications were assessed, mainly *via* Ferumoxytol – a ultrasmall superparamagnetic iron oxide nanoparticles (USPION) formulation – and all of them were discontinued or did not provide acceptable outcomes (U.S. National Library of Medicine, 2013, 2014a,b). Indeed, as far as we know, the overall application of this kind of nanoparticles for MRI did not obtained the expected results and Ferumoxytol is now only used as iron replacement therapy for deficiency anemia in adult patient with chronic kidney diseases (Bobo et al., 2016).

Consequently, despite the long-dated use of inorganic nanoparticles in nanomedical applications, relatively few examples were reported for cardiac regeneration purposes and efficient clinical translation is still missing.

## Nanomedicine in Cardiac Regeneration: Where Are We Heading To?

Multi-therapies aiming to combine the regeneration potential of undifferentiated cells, the *in situ* reprogramming of cardiac fibroblasts and the simultaneous release of drugs appear promising, especially when fueled by the potential of cell-based therapies. Indeed, the use of different smart nanomaterials in combination with other technologies may lead to the development of advanced therapeutic strategies, which may strengthen the applicability of nanomedicine in the treatment of cardiac diseases.

As an example, hiPSC-derived cardiomyocytes (hiPSC-CMs) were recently combined with injectable nanostructured hydrogels loaded with erythropoietin (EPO), resulting in reduced cell death and increased remodeling post-MI (Chow et al., 2017). Also, injectable biomaterials have been used as stand-alone scaffolds for promoting endogenous repair or delivering therapeutics such as cells, growth factors or small molecules.

In this context, Nguyen et al. (2015) used matrix metalloproteinase (MMP)-responsive hydrogels that displayed the ability to be retained at the infarcted site upon enzymatically triggered bio-transformation, thus being potentially suitable for the sustained delivery of therapeutic molecules. Another recently developed strategy called *THEREPI* relies on the use of a biocompatible patch, which is placed epicardially at the border zone of the infarcted heart to achieve the sustained delivery of drugs, macromolecules and possibly cells for cardiac therapy (Whyte et al., 2018). Ideally, *THEREPI* can be efficiently used for the *in situ* administration of therapeutic nanoparticles, thus increasing their retention at the diseased site and improving cargo delivery.

In the case of ischemic cardiomyopathies, improved cargo delivery can be potentially obtained by relying on the enhanced permeability and retention effect (EPR). Similar to the blood vessels originated during tumor development, also those formed at the initial stages after MI are typically aberrant, marked by capillary sprouting, excessive vessels branching, abnormal levels of endothelial cell proliferation, distorted and enlarged vessels, resulting in weak and leaky vasculature (Paulis et al., 2012; Lundy et al., 2016). For those reasons, EPR phenomenon has been exploited for promoting the targeting of NPs at the diseased area, limiting their accumulation in healthy tissues (Golombek et al., 2018). However, while EPR occurring at tumor sites has shown poorly reproducible results, thus jeopardizing the applicability of NPs to cancer therapy (Danhier, 2016), the same phenomenon has been proven to be stable and reproducible following MI in the heart (Weis, 2008). Although mature fibrotic scar is known to be poorly vascularized (van der Meel et al., 2017) the exploitation of EPR phenomenon in the heart, soon after infarction, appears as a promising approach to be translated to the clinics in future for MI treatment (Kalyane et al., 2019).

Besides the pursue of innovative materials and strategies for enhancing therapeutics delivery, the investigation of new pathways involved in cardiac homeostasis is of utmost importance, due to the possibility to target their components for ultimately improving therapeutic outcomes, hence delaying or reversing cardiac dysfunction. In particular, the modulation of cardiac metabolism, gene expression, pharmacological therapy and miRNA-mediated regulatory network represent new and appealing opportunities for the treatment of cardiovascular diseases (Rochette et al., 2015).

In this direction, Hippo pathway has emerged as a possible switch in cardiomyocyte proliferation (Torrini et al., 2019), being tightly connected to the onset and progression of cardiomyopathies (Clippinger et al., 2019), as explained above. Its specific manipulation in the contractile figures of the heart may become a novel therapeutic option for treating cardiac diseases. Nevertheless, to the best of our knowledge, the only NP-based formulation for targeting YAP is at present represented by siRNA-lipid nanoparticles for silencing the protein expression in hepatocellular carcinoma cells and promote tumor regression (Fitamant et al., 2015). Therefore, research for developing NPs able to modulate the YAP activity in cardiomyocytes is at its infancy and may revolutionize the treatment of cardiac diseases and the applicability of NPs in the near future.

Furthermore, it is worth to highlight the potential held by gene editing in the restoration of cardiac function. The discovery that the clustered regularly interspaced short palindromic repeats (CRISPR)/CRISPR-associated (Cas) system could be used to introduce sequence-specific dsDNA cleavage in human cells has revolutionized the research worldwide (Jinek et al., 2012). CRISPR is involved in bacteria and archaea’s adaptative immune system against viruses and their engineering for biological applications has enabled their application in different areas of investigations (Doudna and Charpentier, 2014). Nanomedicine-related sciences are of course included in this development and different systems have been engineered for carrying CRISPR/CAS9 machinery components and guide the genetic reprogramming inside the cells, based on lipid and inorganic nanoparticles (Lee et al., 2017; Liu et al., 2019). Therefore, although the exploitation of this technique in nanomedicine applied to cardiac regeneration can be attractive in the case of genetically-determined cardiomyopathies, technical challenges connected to its specificity must be considered. Indeed, while few studies have already shown that cardiomyocytes can be edited in the post-natal murine heart by CRISPR/Cas9 system components, the efficiency and safety of this strategy is still far from being characterized (Carroll and Olson, 2017). Moreover, the carriage of multiple components (Cas9 ribonucleoprotein, donor DNA and guide RNA) required for this therapy need complex nanoparticle-based systems, hardly scalable and possibly expensive.

Finally, in developing new therapies it is important to consider more practically physiological-like tissue models, not only for a more effective *in vitro* to *in vivo* transition of pharmacological studies, but also for disease modeling and studying the potential toxicity of nanomaterials. Traditional pre-clinical screenings are either made with monolayer cells on top of two dimensional (2D) and often rigid substrates, or in animal models which may not always reflect the human physiology precisely. Instead, a promising strategy to overcome these limitations involves utilizing organ-on-a-chip technologies, where recent microfluidic advances are combined with complex three-dimensional (3D) cell biology that provides organ-like physiology and pathophysiological cellular and tissue level responses (Ergir et al., 2018; Rothbauer et al., 2019).

## Conclusion

Despite any progresses based on healthier life styles, cardiovascular diseases remain the major cause of death globally, according to the WHO (World Health Organization, 2019). Therefore, the development of new therapies to induce cardiac protection and repair are required to help reducing undesirable drugs’ side effects and ultimately improving the life quality of the patient.

In this scenario, material scientists need to exploit the physico-chemical properties of nanomaterials to develop suitable nanotools for smart delivery, targeting proteins and pathways involved in cardiomyocytes protection, differentiation and/or proliferation. Notwithstanding the great progresses made in the direction of a clearer comprehension of the nanoparticles behavior in biological environments, major challenges remain (Heath, 2015). The bioavailability, accumulation at the desired site and efficient release of the therapeutic cargo are just a small part of the challenges nanodrugs must face once administered to the body.

The poor understanding of the biological barriers, the misinterpretation of drug delivery concepts, the cost-effectiveness, manufacturing, scaling up, and regulatory issues have affected the clinical translation of nanomedicine so far, as well as its application for cardiac regeneration purposes (van der Meel et al., 2017).

Different NP-based systems were developed aiming to restore heart function. However, it is still difficult to deduct general guidelines describing the type of material, the class of drugs, the targeting strategies that may be more promising for the given purpose. The successes liposomes obtained in clinical outcomes and in different applications, make them suitable candidates for the treatment of cardiac diseases. However, it is undeniable that polymeric materials display higher tunable properties combined to superior capability of accommodating cargos.

Therefore, nanotechnologists, together with cell/molecular biologists and clinicians, have the duty of finding a common ground with pharmaceutical companies in order to bring potential therapeutic nanomedical devices for cardiac regeneration closer to their clinical translation.

## Author Contributions

MC proposed the subject and conceived the general structure of the review. MC and SF revised the existing literature regarding nanomedicine and the use of nanoparticles for cardiac regeneration. JV revised the literature on miRNAs involved in cardiac development and repair. EE revised the literature on cardiac diseases biology. MC and SF wrote the manuscript. FC and GF revised the text and contributed to the discussion and conclusion.

## Conflict of Interest

The authors declare that the research was conducted in the absence of any commercial or financial relationships that could be construed as a potential conflict of interest.
